# Precocious Torpor in an Altricial Mammal and the Functional Implications of Heterothermy During Development

**DOI:** 10.3389/fphys.2019.00469

**Published:** 2019-04-24

**Authors:** Fritz Geiser, Jing Wen, Gansukh Sukhchuluun, Qing-Sheng Chi, De-Hua Wang

**Affiliations:** ^1^State Key Laboratory of Integrated Management of Pest Insects and Rodents, Institute of Zoology, Chinese Academy of Sciences, Beijing, China; ^2^Centre for Behavioural and Physiological Ecology, Zoology, University of New England, Armidale, NSW, Australia; ^3^Mammalian Ecology Laboratory, Institute of General and Experimental Biology, Mongolian Academy of Sciences, Ulaanbaatar, Mongolia; ^4^University of the Chinese Academy of Sciences, Beijing, China

**Keywords:** Asia, desert hamsters (*Phodopus roborovskii*), heterothermy, altricial, body temperature, metabolic rate, torpor

## Abstract

Most mammals and birds are altricial, small and naked at birth/hatching. They attain endothermic thermoregulation at a fraction of their adult size at a vulnerable stage with high heat loss when many could profit from using torpor for energy conservation. Nevertheless, detailed data on the interrelations between torpor expression and development of endothermic thermoregulation are currently restricted to <0.1% of extant endotherms. We investigated at what age and body mass (BM) desert hamsters (*Phodopus roborovskii*), wild-caught in Inner Mongolia and born in autumn/early winter when environmental temperatures in the wild begin to decrease, are able to defend their body temperature (T_b_) at an ambient temperature (T_a_) of ∼21°C and how soon thereafter they could express torpor. Measurements of surface temperatures via infrared thermometer and thermal camera show that although neonate hamsters (BM 0.9 ± 0.1 g) cooled rapidly to near T_a_, already on day 15 (BM 5.5 ± 0.2 g) they could defend a high and constant T_b_. As soon as day 16 (BM 5.8 ± 0.2 g), when their maximum activity metabolism (measured as oxygen consumption) approached maxima measured in vertebrates, animals were able to enter torpor for several hours with a reduction of metabolism by >90%, followed by endothermic arousal. Over the next weeks, torpor depth and duration decreased together with a reduction in resting metabolic rate at T_a_ 30–32°C. Our data show that development of endothermy and torpor expression in this altricial hamster is extremely fast. The results suggest that precocious torpor by juvenile hamsters in autumn and winter is an important survival tool in their vast and harsh Asian desert habitats, but likely also for many other small mammals and birds worldwide.

## Introduction

Hibernation (multiday torpor) and daily torpor are used by many endothermic mammals and birds to reduce energy expenditure, often to deal with a shortage of food or adverse environmental conditions (Boyer and Barnes, 1999; Nowack et al., 2017). Torpor in these “heterothermic endotherms” is characterized by a controlled reduction of body temperature (T_b_) from high normothermic values, typically ranging from about 36 to 41°C, to values usually between 0 and 29°C and a substantial decrease of metabolic rate (MR) by up to 99% (Boyer and Barnes, 1999; McKechnie and Mzilikazi, 2011; Ruf and Geiser, 2015; Dausmann and Warnecke, 2016).

Torpor expression is strongly affected by size. Torpor is more common in small than in large endotherms and, apparently to maximize energy savings during the torpid state, small species have on average lower minimum T_b_s and their MR reductions are more pronounced (Ruf and Geiser, 2015). This seems to be to a large extent related to the fact that, when normothermic, small species have to compensate for high heat loss via a relative large surface area, have a limited capacity for fat storage and high costs for locomotion (Tucker, 1975; Calder, 1996). Moreover, because the lower critical temperature of the thermo-neutral zone (TNZ) of small species is often around 25 to 30°C and ambient temperature (T_a_) experienced in the wild is generally below that, they typically have to produce heat endogenously because of the large T_b_–T_a_ differential (Tattersall et al., 2012; Kronfeld-Schor and Dayan, 2013; Riek and Geiser, 2013; Withers et al., 2016; Mitchell et al., 2018).

Although almost all mammals and birds are competent endotherms as adults, the vast majority are altricial at birth or hatching. Altricial neonates or hatchlings are small, naked and uncoordinated and, when removed from the nest or away from their parents, cool rapidly to near ambient temperature (T_a_) because they are only partially endothermic and unable to produce sufficient endogenous heat for maintenance of a high and constant T_b_ (Dawson and Evans, 1960; Morrison and Petajan, 1962; Holloway and Geiser, 2000); therefore they are often referred to as being poikilothermic (Greek “poikilo” = variable, “therme” = heat). As these animals grow to larger size and fur or feathers develop together with a maturation of the nervous and other organ systems, the ability for endogenous heat production and heat retention improves and eventually they are able to maintain a constant high T_b_, at least during exposure to moderate T_a_s. These newly endothermic young, typically at about 20–50% of adult body mass, are highly vulnerable to heat and energy loss or even death by starvation when they maintain a high constant T_b_ at times the parents are unable to supply enough food and because of their inexperience in obtaining food on their own (Geiser, 2008).

Potentially, using torpor during periods of high heat loss and insufficient food supply would provide an avenue to enhance survival of young. However, although such energetic bottlenecks are likely faced occasionally or even frequently by the majority of small young mammals and bird species, as for example during bad weather, information on torpor during development is limited to only a few species (<15 species or <0.1% of the >15,000 mammals and bird species), including some small marsupial mammals, a few placental mammals, and a few birds (Nagel, 1977; Geiser, 2008; Eichhorn et al., 2011; Giroud et al., 2014; Wacker et al., 2017). For most of the studied species the rate of development is rather slow, and, especially in marsupials, for which most detailed data are available, first expression of torpor is observed only months after birth following a prolonged poikilothermic phase (Geiser et al., 2006, 2017; Wacker et al., 2017). The slow development in many species expressing torpor is supporting the view that heterothermic species often have a slow life history (Turbill et al., 2011), typically producing only 1 or 2 litters/young per year.

The purpose of our investigation was to provide the first data on the development of endothermy and torpor expression in small desert hamsters (*Phodopus roborovskii*; adult BM ∼25 g). This species is interesting with regard to its reproductive and developmental biology as it differs from most other species in which development of torpor has been investigated. It produces up to four litters of up to ten young per year, can breed within 2 months of birth, lives for only 1 or 2 years in the wild, and is clearly r-selected (Kolynchuk, 2015). Desert hamsters are distributed over the harsh deserts of continental Asia and, as adults, use daily torpor to aid survival in their environment (Chi et al., 2016). As they reproduce well into autumn, their young are potentially exposed to adverse environmental conditions and energy shortages in late autumn/early winter, and may require the use of energy savings mechanisms such as torpor to survive over winter into adulthood. We therefore tested the hypothesis that this fast-growing species is able to express torpor early during the growth phase, soon after endothermy is attained. Such data will improve the understanding of the functional significance of torpor especially in rapidly developing altricial mammals and the role of heterothermy during development in endotherms in general.

## Materials and Methods

### Animals

Litters of five female desert hamsters, captured in Inner Mongolia (Hunshandake sandy land, 43°11 ′N, 116°10 ′E) on the last week of August 2017, were used in our study. They were transported to the Chinese Academy of Sciences in Beijing on 1 September, where they were held individually in standard cages supplied with bedding (wood shavings and cotton wool), food (mouse pellets: crude protein ≥18%; crude fat ≥4%; coarse fiber ≤5%; ash ≤8%; moisture ≤10%, Beijing HFK Bio-Technology Co., Ltd., and Millet seeds) and water *ad libitum* under a LD 12:12 photoperiod and T_a_ 21 ± 1°C. This T_a_ was selected for maintenance and measurements because during the trapping month mean maximum T_a_ was 23.9 ± 3.1°C and the mean minimum T_a_ was 12.2 ± 3.2°C (China Meteorological Data Service Center) and because we wanted to ensure that the animals were not stressed, but nevertheless experienced a mild cold load during experiments. This study was carried out in accordance with the recommendations of Animal Care and Use Committee of Institute of Zoology, the Chinese Academy of Sciences. The protocol was approved by the Animal Care and Use Committee of Institute of Zoology, the Chinese Academy of Sciences.

**FIGURE 1:**
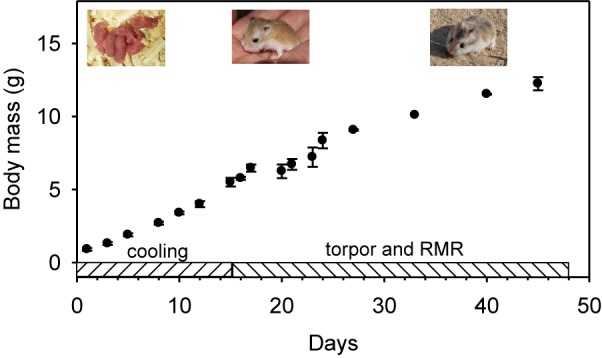
Experimental time-course and mean body mass with SD and pictures of developing desert hamsters at different ages.

Two females gave birth on 4 September (Pr1, 5 young; Pr2, 4 young). These individuals were used to determine rates of growth, rates of cooling from day 1 to day 15, torpor expression from day 16 to day 45, and resting metabolic rate (RMR) (Figure 1). Two females (Pr3 and 4) gave birth to litters of 5 young each on 7th September; 9 of these animals were used to measure RMR and torpor expression. An additional female (Pr 5) mated in captivity and gave birth to a litter of 5 young in December; 3 of these animals were also used to measure RMR and torpor expression. A total of 20 young were examined.

### Cooling Experiments

Rates of cooling in the two litters born on 4 September were measured from day 1. For these measurements, animals were removed from their nests and placed individually into cardboard beakers at the same T_a_ as in the holding cage (T_a_ 21 ± 1°C) and their surface temperature (T_s_) measured non-invasively using two approaches. Maximum T_s_ was measured ventrally on naked skin early and on sparsely fur covered skin later during the development to the nearest 0.1°C with an infrared thermometer (Digitech, QM-7218) at the time of removal from the nest and then at 10 min intervals over the next 30 min, during which time the animals were not touched. Thermographs (Flir Model C2, resolution 0.1°C; pre-calibrated by manufacturer) were taken at the same time of the T_s_ readings. Emissivity was set at 0.95 for both infrared thermal devices throughout the study. Readings from thermometer and thermal camera used here were compared with axillary temperatures measured under the foreleg of the same young hamsters to the nearest 0.1°C using a calibrated thermocouple probe (TES-1310, TES Electric Electronic Corp.); these measurements were within 0.5°C of T_s_ reading. Animals were weighed to the nearest 0.1 g with an electronic balance at the end of the cooling experiments and were returned to their nest.

### Torpor Induction

#### Metabolic Trials

From 1 day after animals were able to maintain a constant T_b_ at T_a_ 21 ± 1°C over 30 min, we tested whether they were able to enter a bout of torpor for energy conservation after food restriction. For these measurements, juveniles were removed from the nest in the late afternoon and placed individually into a respirometry chamber (volume 470 ml) to measure MR as the rate of oxygen consumption overnight (for up to 22 h) with an open flow respirometry system (Sable Systems, TurboFOX Complete Field System, including a mass-flow meter). An incubator (Yiheng Model LRH-250, Shanghai, China) was used to maintain constant T_a_ within the respirometry chamber during the measurement intervals at T_a_ 22.0 ± 0.5°C. Fresh air from outside was pumped through the chamber at ∼150 ml/min. Before entering the chamber, air passed through a copper coil to ensure that its temperature was adjusted to that inside the incubator. After passing through the respirometry chamber, the gas was subsampled and dried using a non-chemical gas drier (Sable systems, ND-2); approximately 70 ml/min at a stable flow rate was analyzed and recorded every 15 s. Baseline measurements of reference outside air were carried out every 3 h to compensate for potential drift of the oxygen sensor during long-term metabolic rate monitoring. Resting metabolic rate was taken as an average of 10-min the lowest, constant and stable readings during the first 3 h while hamsters were normothermic and in their resting phase.

#### Resting Metabolic Rate Measurements in the TNZ

From the age of 17 days (BM 6.0 g), resting metabolic rate of individuals was also measured at T_a_ 30–32°C (T_a_ 25–33°C, is the thermal neutral zone, TNZ, of adult desert hamsters; Zhan and Wang, 2004) for at least 3 h using the same respirometry equipment described above from day 17 until 45 days. In addition, after day 45, 9 adult hamsters from different litters that were 8–10 months old and were born in our laboratory colony with BM ranging from 19 to 26 g were examined. The lowest consecutive values over 10 min were assumed to be the RMR. Hamsters were weighed to the nearest 0.1 g before and after each respirometry measurement. The average of the two values of body mass was used to calculate the mass-specific metabolic rate.

### Torpor Determination

In the morning when the MR of juvenile hamsters indicated that they were in a state of torpor (MR well below 75% of RMR measured in the same individual at the same T_a_; Hudson and Scott, 1979; Chi et al., 2016) animals were removed from the chamber and their thermograph was taken and/or their T_s_ was measured immediately as described above. A T_s_ of ≤29.6°C was considered as an alternative definition of torpor because the mean resting T_s_ before measurements was 34.6 ± 0.6°C and a fall of T_b_ by 5°C or more from the resting T_b_ is often used for defining torpor (Ruf and Geiser, 2015). Animals were handled with gloves to ensure there was no heat transfer from the hand and then returned to the respirometry chamber within 1 min to determine whether they could rewarm from torpor; these measurements were repeated during the arousal process. Torpor duration was defined as the time with MR below 75% of RMR at the same T_a_ (Chi et al., 2016). Some young hamsters were placed individually in cages on sawdust into the incubator at T_a_ 22.0 ± 0.5°C overnight and it was determined via T_s_ and thermograph measurements whether they could enter and arouse from torpor. To minimize premature termination of torpor bouts measurements of T_s_ were conducted when obvious movements of a hamster were observed, which often happens before they arouse. Nevertheless, as some torpor bouts were disrupted, torpor bout duration was not further analyzed.

### Statistics

Statistical test were performed using R (Pinheiro et al., 2018) and SPSS, Version 17.0. Linear mixed models were used with litter and animal ID as random factors to examine whether T_s_ in growing hamsters is related to BM and age because the same individuals were measured. Repeated Measures ANOVAs were conducted on T_s_ measurements both before and after the cooling experiments as well as on T_s_ and body mass among different ages together with LSD multiple comparisons. One sample t tests were used to examine the difference between T_s_ and T_a_. Linear mixed models using litter as a random factor were used to analyze the relationship between RMR and BM. *P*-values of <0.05 were considered statistically significant. Numeric values are provided as means ± SD for the number of animals “n” measured.

## Results

### Body Mass in Young Hamsters During Cooling Experiments

The body mass (Figure 1 and Table 1) of neonate hamsters (litters of Pr1 and Pr2) ranged from 0.8 to 1.1 g (0.9 ± 0.1 g) and the animals were pink and naked. On day 5, mean body mass was 1.9 ± 0.1 g, increased to 2.7 ± 0.1 g on day 8, to 3.4 ± 0.1 g on day 10, to 4.0 ± 0.2 g on day 12 and to 5.5 ± 0.3 g on day 15 when hamsters looked like small adults with dorsal fur covering (Figure 1), but the ventral fur was still thin and sparse.

**Table 1:** Maximum T_s_ readings from infrared thermometer and thermographs in developing desert hamsters before (0 min) and after 30-min cold exposure at T_a_ 21°C.

Age (day)	Body mass (g)	Infrared thermometer		Thermal camera	
			
		T_s_ 0 min (°C)	T–_s_ 30 min (°C)	T_s_ 0 min (°C)	T–_s_ 30 min (°C)
1	0.9 ± 0.1^a^	32.3 ± 1.0^a^	21.5 ± 0.3^a^	33.2 ± 1.3	23.1 ± 0.6^a^
5	1.9 ± 0.1^b^	32.9 ± 0.7^ab^	24.1 ± 0.2^b^	34.2 ± 1.0	26.6 ± 0.4^b^
8	2.7 ± 0.1^c^	33.5 ± 0.6^b^c	25.1 ± 0.4^c^	34.7 ± 0.7	28.2 ± 0.4^c^
10	3.4 ± 0.1^d^	34.1 ± 0.6^d^	26.3 ± 0.7^d^	34.7 ± 0.6	28.5 ± 1.3^d^
12	4.0 ± 0.2^e^	34.0 ± 0.5^c^d	30.0 ± 2.2^e^	34.0 ± 0.4	31.6 ± 2.3^e^
15	5.5 ± 0.3^f^	33.3 ± 0.4^ab^	34.2 ± 0.7^f^	33.9 ± 0.7	35.0 ± 0.7^f^


*All values are means with SD for *n* = 8 individuals from 2 litters. Different letters in the same vertical row indicate significant differences among ages. RMANOVA, for body mass, *F*_*5,35*_ = 491.0 and *P* < 0.001. For T_*s*_ measured after cold exposure of 0 or 30 min by using infrared thermometer, *F*_*5,35*_ = 6.73, *p* < 0.001 and *F*_*5,35*_ = 997.7, *p* < 0.001, respectively. For T_*s*_ measured after cold exposure of 0 or 30 min by using infrared thermal camera, *F*_*5,35*_ = 3.25, *p* = 0.072 and *F*_*5,35*_ = 103.2, *p* < 0.001, respectively.*

### Cooling Experiments

**FIGURE 2:**
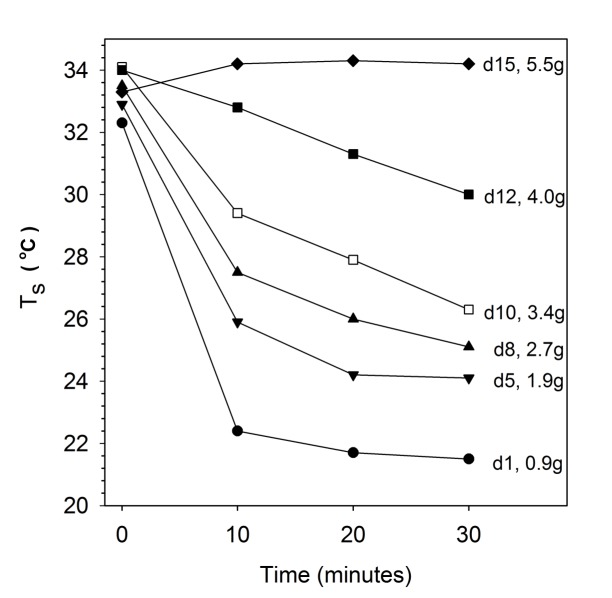
Cooling rates of desert hamsters at different ages from day 1 (d1) to day 15 (d15) at T_a_ 21 ± 1.0°C with corresponding body masses. Note the fast decline of surface temperature (T_s_) measured with infrared thermometer on day 1 and the reduction of cooling with age. Only means are shown for clarity, SD are provided in Table 1.

Rates of cooling in hamsters on day 1 were rapid (Figure 2 and Table 1). The T_s_ fell from 32.3 ± 1.0°C to ∼22°C within 10 min and after 30 min the T_s_ of 21.5 ± 0.3°C was barely distinguishable from the T_a_ of 21.2°C (*t* = 2.871, *df* = 7, *p* < 0.05). On day 5, initial T_s_ was slightly raised to 32.9 ± 0.7°C and cooling was slowed, with animals maintaining a T_s_–T_a_ differential of 2.1 ± 0.2°C at 30 min and animals showed slight shivering (*t* = 25.31, *df* = 7, *p* < 0.01). Initial T_s_ further increased and cooling was further slowed on day 8, and 10 and 12 when animals showed good coordination and some shivering, but the eyes were still partially closed. On day 15 the initial T_s_ of 33.3 ± 0.4°C was actually lower, than the T_s_ of 34.2 ± 0.7°C measured after 30 min when the mean the T_s_–T_a_ differential was 12.4°C; these animals showed strong shivering, digging and grooming, had stored millet seeds in their cheek pouches and tried to bite. The T_s_ at the beginning of cooling experiments were similar, but differed substantially after 30 min (Figure 2 and Table 1).

**FIGURE 3:**
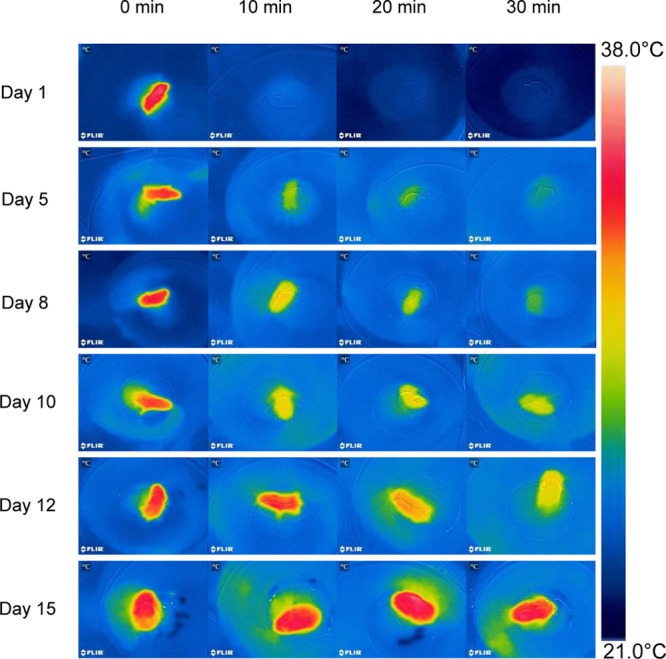
Thermographs of a representative desert hamster at each age experienced a cold exposure at T_a_ 21°C during development. Note the similar surface temperature (T_s_) at 0 min, the different T_s_ after 30 min, the rapid fall of T_s_ on day 1, and the maintenance of a high T_s_ on day 15.

Thermographs further emphasize the rapid developmental change in thermoregulatory ability (Figure 3 and Table 1). In 1 day old hamsters the maximum T_s_ was 35.6°C at 0 min (mean 33.2 ± 1.2°C), decreasing to 23.1 ± 0.6°C in 30 min. While the maximum T_s_ at 0 min seemed to change slightly because of the increasing fur cover it did not differ significantly with age (*F*_5,35_ = 3.25, *p* = 0.072). However, the rate of cooling slowed with growth as indicted by the thermograph color changes (Figure 3 and Table 1) and after 30 min exposure, T_s_ increased steadily to a maximum of 35.4°C on day 15. The maximum T_s_ after 30 min cold exposure was strongly affected by age and body mass (*F*_5,35_ = 103.2, *p* < 0.001, T_s after cold EXP_ = 0.80 age+22.0, *R*^2^ = 0.88, *P* < 0.001; T_s after cold EXP_ = 2.73 body mass+18.47, *R*^2^ = 0.92, *P* < 0.001). This supports the data from the infrared thermometer measurements that at the age of 15 days, desert hamsters are competent thermoregulators when exposed for short periods to a mild cold load.

### Metabolic Trials

**FIGURE 4:**
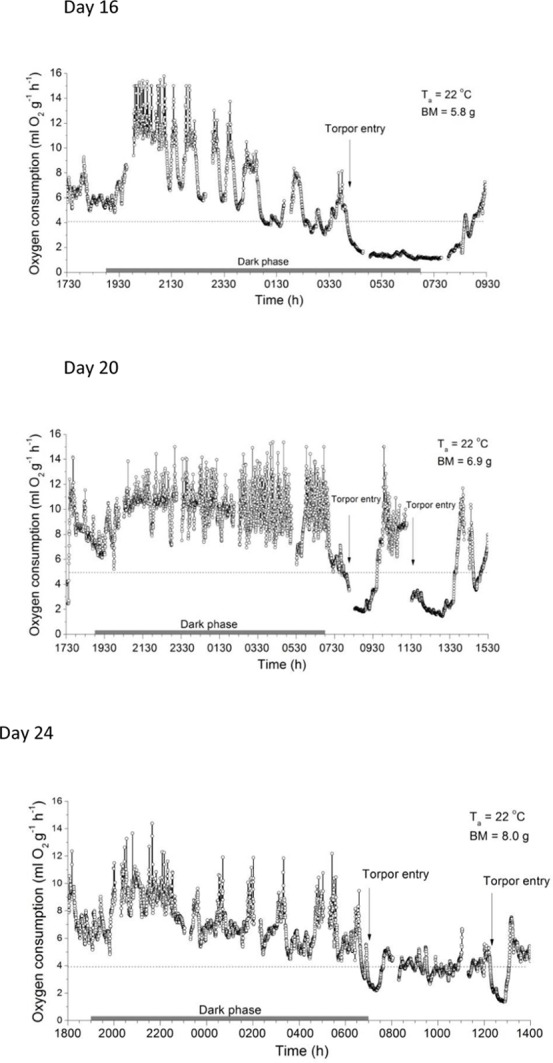
Metabolic rates (MR) of desert hamsters measured as oxygen consumption overnight. Note the high values during activity at night and the rapid fall of MR during torpor entry late at night or early in the morning. Torpor duration and depth decreased with age and increasing size. Gaps in the data points show baseline readings of the respirometry system every 3 h. Horizontal dotted lines show 75% of the resting metabolic rate measured at T_a_ 22°C.

When one of these animals (Pr2 #1) was exposed to T_a_ 22°C overnight on day 16 (mean BM 5.8 g), its MR initially ranged between ∼5 and 9 ml O_2_ g^-1^h^-1^, indicating resting and slight activity (Figure 4). Soon after the lights went off, MR increased to activity maxima approaching 16 ml O_2_ g^-1^h^-1^, interrupted by apparent resting phases. Both the maxima and minima declined with time in the dark phase until entry into torpor occurred at around 0400 h. Entry into torpor was characterized by a rapid fall of MR to a minimum MR during torpor of 1.14 ± 0.04 ml O_2_ g^-1^h^-1^ (∼30% of RMR at T_a_ 22°C, ∼7% of that during activity). When removed from the chamber after a torpor bout of ∼4 h at 0820 h, the T_s_ measured by infrared thermometer was 25.0°C, similar to that measured with the thermal camera.

Two other 16 day old individuals (Pr1 #2 and #3, BM = 5.8 ± 0.2 g) exposed in separate cages on wood shavings overnight without food and water, but without MR measurements, initially had a T_s_ of 34.4 ± 0.4°C, which fell to 24.6 and 25.8°C at 0820 h, and both were able to endogenously rewarm at T_a_ 22°C and increased T_s_ to ∼34°C.

When overnight MR measurements at T_a_ 22°C were repeated between 17 (BM = 6.5 ± 0.3 g) and 45 days (BM ∼12 g) other individuals also expressed torpor (Figure 4), but torpor duration and depth decreased with growth and age (Table 2). The T_s_ of all torpid individuals during or without metabolic rate measurements was 25.1 ± 0.6°C (16 days, BM = 5.8 ± 0.2 g, torpor use in 3/3), 28.8 ± 0.3°C (17 days, BM = 6.5 ± 0.3 g, torpor use 2/3), and 25.2 ± 3.9°C (20 days, BM = 6.3 ± 0.5 g, torpor use 3/3). From 21 days only shallow torpor was observed and usually only 1 out of three measured individuals expressed torpor.

**Table 2:** Torpor metabolic rates (TMR), resting metabolic rates (RMR, maximum torpor duration) and T_s_ in desert hamsters fasted overnight at T_a_ of 22°C.

Animal No.	Age (days)	Body mass (g)	RMR (mlO_2_ g^-1^h^-1^)	TMR (mlO_2_ g^-1^h^-1^)	Torpor duration (min)	Lowest T_s_ (°C) after fasting	T_s_ (°C) before fasting
Pr2 #1	16	6.0-5.5	5.43	1.14	255	25.0	34.0
Pr1 #1	16	6.1-5.2				24.6	34.4
Pr1 #2	16	6.4-5.4				25.8	34.8
Pr5 #1	17	7.5-6.1	5.64	2.28	83	28.6	34.2
Pr5 #2	17	7.2-5.6				30.6	34.4
Pr5 #3	17	6.9-5.5				29.0	34.8
Pr3 #1	20	7.3-6.5	6.58	1.64	148	24.0	33.7
Pr4 #1	20	6.3-5.3				29.6	34.8
Pr4 #2	20	6.6-5.5				22.0	34.2
Pr3 #2	21	6.9-5.5	6.08	2.00	136	23.4	34.8
Pr4 #3	21	7.6-6.2				31.6	35.7
Pr4 #4	21	7.8-6.3				32.6	35.8
Pr2 #2	23	8.6-7.3	5.32	1.45	47	32.6	34.0
Pr1 #3	23	7.2-5.5				33.8	34.4
Pr1 #4	23	8.0-6.7				34.0	34.8
Pr3 #3	24	8.5-7.5	5.24	1.89	46	29.6	33.8
Pr3 #4	24	10.1-8.1				29.2	35.4
Pr4 #5	24	8.9-7.0				32.4	33.8
Pr2 #3	27	10.1-8.1	5.53	2.58	15	32.4	34.4
Pr2 #4	27	10.2-8.0				33.2	35.8
Pr1 #5	27	10.0-8.0				32.0	35.6
Pr4 #1	33	11.0-9.2	6.07	3.12	10	32.6	34.4
Pr1 #2	39	12.5-10.6	6.02	2.48	18	32.4	34.0
Pr4 #2	40	12.5-10.5	5.33	2.66	23	33.6	34.4
Pr1 #5	44	13.4-12.0	4.75	2.65	12	33.0	34.4
Pr2 #1	45	12.7-10.9	4.99	3.14	10	32.6	34.2


*All individuals could rewarm from low T_*b*_ via endogenous heat production. Two values of body mass for each individual are shown before and after measurements.*

**FIGURE 5:**
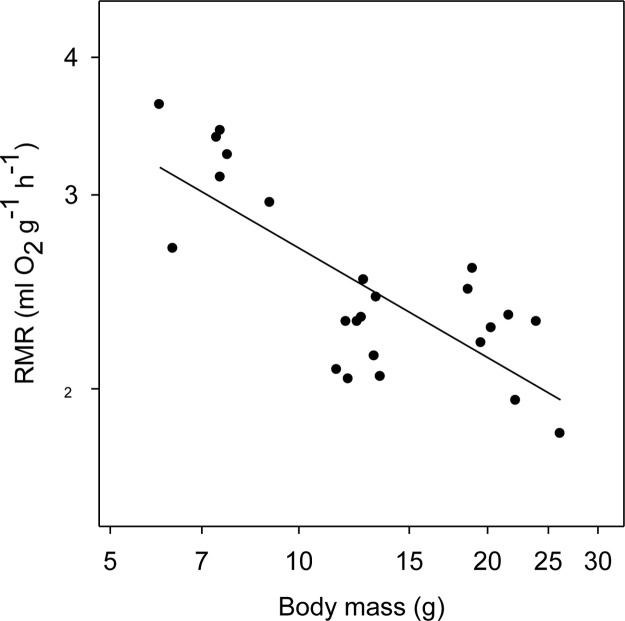
Resting metabolic rate (RMR) of desert hamsters in the TNZ as a function of body mass (BM) measured as rate of oxygen consumption at ambient temperatures (T_a_) of 30 to 32°C. The RMR fell with the increase of BM (log_10_y = 0.757–0.33log_10_x; *r*^2^ = 0.59). RMR data were included for adult hamsters with body mass over 15 g (8–10 months age) raised under same laboratory conditions.

The mass-specific RMR measured in the TNZ at T_a_ 30–32°C ranged from 3.62 ml O_2_ g^-1^ h^-1^ (17 days, BM = 6 g) to 1.82 ml O_2_ g^-1^ h^-1^ (BM = 26.2 g adult). The log_10_ RMR was a negative function of log_10_ BM, with a slope of –0.33 (*F*_1,22_ = 20.89, *P* < 0.001; *R*^2^ = 0.59) (Figure 5).

## Discussion

The rate of development of desert hamsters is astonishing. Endothermy is reached as early as at 15 days of age at a body mass of only 5.5 g and already at 16 days (BM 5.8 g) the animals are able to express torpor with a rapid decline of MR at torpor entry and the ability of rewarming from low T_b_ at the end of the torpor bout.

The development of thermoregulation and morphology was qualitatively similar to what has been observed in other altricial birds and mammals (Morrison and Petajan, 1962; Geiser, 2008). Initially when the animals were small and naked, rates of cooling were high. Rates of cooling decreased with an increase in size, improvement of insulation, but likely also an improvement of non-shivering thermogenesis from brown adipose tissue (BAT) (Oelkrug et al., 2015). The difference between our and most other studies was the speed of development with competent endothermy in desert hamsters reached only after 15 days, much faster than in many other species, especially small marsupials that take several weeks to reach that state (Geiser et al., 2006; Wacker et al., 2017). However, the values were similar that what has been observed in the related and similar-sized Djungarian hamster (*Phodopus sungorus*) (Bae et al., 2003).

The fast development of thermoregulation of desert hamsters was followed by the ability to enter and arouse from torpor at an age as young as 16 days (BM 5.8 g). Although we quantified torpor at a rather mild T_a_ because of consideration of the animals’ wellbeing, lower T_a_s likely would have resulted in increased torpor use (see Chi et al., 2016) especially later during development. Torpor was characterized by a reduction of MR to <10% of the MR during activity, which was as high as that of bats and birds during flight, which are among the highest MR measured for vertebrates (Speakman and Racey, 1991). Rewarming from low T_b_ during torpor was achieved by juvenile desert hamsters although heat production is reduced at low T_b_ and although the high relative surface area at a small BM facilitates heat loss and makes endogenous rewarming difficult (Currie et al., 2015; Wacker et al., 2017). The fast development of torpor differs again from small marsupials, which reach that state only after several weeks (Wacker et al., 2017), but also from the related *P. sungorus*, which expressed fasting-induced torpor only from the age of 28 days (Bae et al., 2003). This difference may appear surprising considering the similar size, similar development of endothermy and similar general biology of the two congeners. However, the discrepancy is likely explained by the commencement of measurements of fasting-induced torpor in *P. sungorus*, which did not begin until day 21, soon after the animals had been implanted with transmitters. Therefore, it is possible that, if *P. sungorus* had been measured earlier, they would be rather similar to desert hamsters, an assumption that is supported by the observation of 2-deoxy-D-glucose (2-DG) induced T_b_ reduction in *P. sungorus* at the age of 16 days (Bae et al., 2003). Although it has been argued that the 2-DG induced reduction in T_b_ differs from natural torpor (Westman and Geiser, 2004, but see Chi et al., 2018) it nevertheless shows that the animals were able to rewarm from low T_b_ at a very early age.

What is the likely function of torpor during development? Constant endothermy in small species is extremely costly because of the high heat loss and requires a more or less uninterrupted supply of energy in the form of food. If adults are unable to supply the food or juveniles unable to obtain it, they are in danger of starvation or even death. This can be overcome by the expression of torpor, which reduces energy and therefore foraging requirements substantially (Nowack et al., 2017), and can prolong the time over which stored energy in the body can be extended, in extreme cases for up to several months (Ruf and Geiser, 2015). Of course huddling in nests also will reduce energy expenditure to some extent and is important during the development (Gilbert et al., 2010). However, it does not prevent occurrence of hypothermia in groups of young in the wild (Holloway and Geiser, 2000). Moreover, the limitation of huddling lies with its inability of reducing MR below basal levels, whereas during torpor MR values well below BMR can be reached and energy loss can be minimized (Namekata and Geiser, 2009). While survival is clearly an important function of torpor during development it also has the potential to be used for diverting nutrients to facilitate growth as growth rate on limited food is enhanced by torpor use (Giroud et al., 2014). Since the vast majority of mammals (for example, marsupials, bats and many rodents) and birds (for example passerines) are altricial at birth/hatching and will have to undergo similar morphological and functional changes described here, the expression of torpor during the development may be far more often employed that is currently appreciated, and may even include some small precocial species.

Past data suggested a different development sequence in torpor expression between birds and marsupial mammals on one hand and placental mammals on the other (Geiser, 2008). In birds and marsupials torpor expression seemed to develop immediately after competent endothermy was attained (i.e., heterothermic endothermy develops immediately after poikilothermy). In contrast, as outlined above, the data on the placental rodents suggested that the initial partial endothermic phase (poikilothermy) is followed by an intermediate homeothermic phase with constant high T_b_ lasting for about 2 weeks in hamsters (*P. sungorus*) with the first bout of torpor after food restriction observed only at the age of 28 days (Bae et al., 2003). More extreme, in juvenile ground squirrels (*Spermophilus saturatus*) this homeothermic phase is prolonged and torpor is expressed only ∼4 months after birth (Geiser and Kenagy, 1990). However, data from our present study with torpor expression in placental desert hamsters only 1 day after reaching endothermy suggest that there may be no generic difference between marsupial and placental mammals and that perhaps the pattern observed in ground squirrels is a reflection of their extreme seasonal thermal biology characterized by homeothermy in spring/summer and heterothermy only from late summer through autumn/winter. Thus, while our data do not support the view that there may be common developmental differences with regard to torpor expression among endothermic classes or sub-classes they nevertheless suggest that it is widely used by many small endotherms.

It is interesting that torpor expression is reduced with growth. Likely this is related to the reduction of mass-specific RMR in thermo-neutrality (Figure 5), which interestingly, despite the young were growing and should have a raised RMR, had a slope of –0.33 as that measured for BMR in fully grown adult mammals (White and Kearney, 2013). However, and perhaps more importantly with regard to our study, the reduced torpor expression with growth was likely caused by the exposure to only a mild cold load (T_a_ 22°C), a reduction on RMR during cold exposure, and a reduced cost of locomotion that decreases with size (Tucker, 1975). Especially the mild T_a_ differs substantially from those experienced by the animals in the wild in winter when they are exposed to very low T_a_ and food is probably scarce. Therefore individuals in the wild likely will express torpor even when they reach adulthood (Chi et al., 2016).

Clearly heterothermy during development provides juveniles with flexible thermo-energetics and consequently a better chance of survival, as is also the case even before birth during reproduction (Willis et al., 2006). Considering how few species have been studied in this regard, it is possible that torpor during development is used by many if not the majority of altricial species. This interpretation is supported by the fact that shallow torpor during development has been observed even in species considered to be homeothermic as adults such as in juvenile storm petrels (*Oceanodroma furcata*) and laboratory rats (*Rattus norvegicus*) (Boersma, 1986; Nuesslein-Hildesheim et al., 1995). It therefore seems an omission that the phenomenon of torpor use during development has not been more widely examined with the aim to improve the understanding of its diversity and function.

## Ethics Statement

This study was carried out in accordance with the recommendations of Animal Care and Use Committee of Institute of Zoology, the Chinese Academy of Sciences. The protocol was approved by the Animal Care and Use Committee of Institute of Zoology, the Chinese Academy of Sciences.

## Author Contributions

FG finished the main parts of the manuscript. Q-SC, JW, GS, and FG conducted the experiments. FG, Q-SC, and D-HW conceived this study.

## Conflict of Interest Statement

The authors declare that the research was conducted in the absence of any commercial or financial relationships that could be construed as a potential conflict of interest.
